# Diagnostic Value and Clinical Application of mNGS for Post-Liver Transplantation Infection: A Cross-Sectional Study With Case Reports

**DOI:** 10.3389/fmicb.2022.919363

**Published:** 2022-07-01

**Authors:** Dong Zhao, Liping Guo, Dongli Lian, Yuchen Gu, Xu Yan, Hongxing Hu, Jing Yuan

**Affiliations:** ^1^Division of Liver Surgery and Organ Transplantation Center, Shenzhen Third People's Hospital, Second Affiliated Hospital, Southern University of Science and Technology, Shenzhen, China; ^2^Department for Infectious Diseases, Shenzhen Third People's Hospital, Second Affiliated Hospital, Southern University of Science and Technology, Shenzhen, China; ^3^Department of Kidney Transplant, Shenzhen Third People's Hospital, Second Affiliated Hospital, Southern University of Science and Technology, Shenzhen, China

**Keywords:** metagenomic next-generation sequencing, liver transplantation, bronchoalveolar lavage fluid, whole blood, pathogen detection

## Abstract

Liver transplantation is widely acknowledged as the only effective treatment for end-stage liver disease, and infection is reportedly an important cause of postoperative death. Clinical use of metagenomic next-generation sequencing (mNGS) to diagnose postoperative infection and successfully guide drug therapy remains rare. This study included patients with infectious complications after liver transplantation from July 2019 to December 2020 and was divided into three groups: pneumonia, unknown fever, and others (including hepatic failure, kidney failure, cirrhosis after LT, and other postoperative complications that predispose to infection). The mNGS sequencing was used to detect microorganisms, and the results were compared with traditional culture. We found that mNGS yielded improved sensitivity over culture (85.19 vs. 22.22%; *p*<0.0001) but lower specificity (35.71 vs. 89.28%; *p*<0.0001). Among the 48 kinds of pathogens detected, the Torque teno virus 22 (15/122) was the most common, followed by Primate erythroparvovirus 1 (13/122). The top four bacteria included *Klebsiella pneumoniae* (*n* = 8), *Enterococcus faecium* (*n* = 5), *Stenotrophomonas maltophilia* (*n* = 4), and *Escherichia coli* (*n* = 4). *Aspergillus fumigatus* was the most common fungus. The bronchoalveolar lavage fluid (BALF) exhibited the highest proportion of positive findings among sample types, with viral, fungal, and bacterial mixed infection being the most common (*n* = 6, 19.35%). Besides, using mNGS for early diagnosis of infection after liver transplantation may effectively prolong patient survival. This is the first study to explore the application value of mNGS and its comparison with traditional culture in pneumonia and other infections in post-liver transplantation patients. The simultaneous application of these two methods suggested that the Torque teno virus 22, *Klebsiella pneumoniae*, and the *Aspergillus fumigatus* are the most common pathogens of viruses, bacteria, and fungi after LT, suggesting that these pathogens may be associated with postoperative pathogen infection and patient prognosis. The mNGS technique showed distinct advantages in detecting mixed, viral, and parasitic infections in this patient population. Further studies are warranted to systematically elucidate the dynamic evolution and molecular characteristics of infection after liver transplantation.

## Introduction

Liver transplantation (LT) is well-established as the only effective treatment for end-stage liver disease. Since Prof. Starzl performed the first liver transplantation operation in human history in 1963, the expertise for liver transplantation has spread globally (Vonkaulla, 1963). Nowadays, LT has become a routine operation associated with a 1-year survival rate of 82% in the European Liver Transplant Registry and 87% in the Japanese registry, and a 10-year survival rate ranging from 53 to 76% in the American, European, and Japanese registries (Zhang et al., 2021). With refined liver transplantation techniques and the standardization of perioperative management, the postoperative survival rate of LT recipients in China has gradually increased. A study on the medical quality of LT in China in 2018 showed that the mortality rate within 1 week after LT decreased from 3.7% in 2015 to 2.2% in 2018, and the 3-year cumulative survival rate of LT recipients with benign end-stage liver disease was 78.5%, which is excellent according to international standards (Chinese College of Transplant Doctors, and Division of Liver Transplantation Branch of Organ Transplantation, 2019).

Current evidence suggests that infectious complications are significant causes of morbidity and mortality after LT, despite advances in surgical technique, post-transplant care, hospital environments, immunosuppression, infectious disease treatment, infection prevention, and prophylaxis in LT recipients (Cross et al., 2008). An increasing body of evidence suggests that LT recipients are more likely to develop bacterial infections than other transplant recipients, given the complexity of the surgical procedure, which includes penetration of the hepatobiliary system (Kim, 2014). Interestingly, pneumonia is one of the most common infections post-LT, occurring in 8–23% of patients (Angarita et al., 2017). In this regard, a study (El-Badrawy et al., 2017) reported a mortality rate of 75% for post-LT pulmonary infection, the leading cause of early postoperative death. Better understanding and accurate detection of pathogenic microorganisms associated with pulmonary infections are of utmost importance to improve outcomes and reduce morbidity and mortality of post-LT patients.

Indeed, early, rapid, and accurate diagnosis of pathogens is key to treating infections after LT. In the past, pathogen detection was traditionally achieved by microbial morphological culture or nucleic acid detection, associated with long detection periods and low positive rates. In recent years, unbiased metagenomic next-generation sequencing (mNGS) has been used in medical microbiology as an emerging and powerful technique due to its low cost, rapid turnaround time, and convenience. mNGS can generate millions to billions of DNA/RNA sequences per run, enabling metagenomic analysis, and providing a significant advantage over traditional Sanger sequencing (Guan et al., 2016; Fang et al., 2020; Hu et al., 2021; Zhang et al., 2022). Given that next-generation metagenomic sequencing is associated with higher sensitivity (mNGS; Chen et al., 2020; Duan et al., 2021) and is less affected by prior antibiotic exposure, it has been used to diagnose infections after solid organ transplantation. Overwhelming evidence substantiates that mNGS effectively detects pathogens after lung transplantation (Liu et al., 2020; Lian et al., 2021). There is no doubt that mNGS technology can play an essential role in rapidly diagnosing post-LT infections, especially for uncommon pathogen infections. Nonetheless, given the complexity of postoperative infection after LT, the use of mNGS to diagnose postoperative infection and successfully guide drug therapy is rare during clinical practice.

Here, we provide a comprehensive analysis of an mNGS-based approach for rapid detection and identification of pathogenic microorganisms for pneumonia or other infections in patients after LT, especially in postoperative cases of fever of unknown origin. Moreover, we reported cases where mNGS assisted clinical diagnosis and medication to improve the patient's condition.

## Materials and Methods

### General Clinical Data

**Figure 1:**
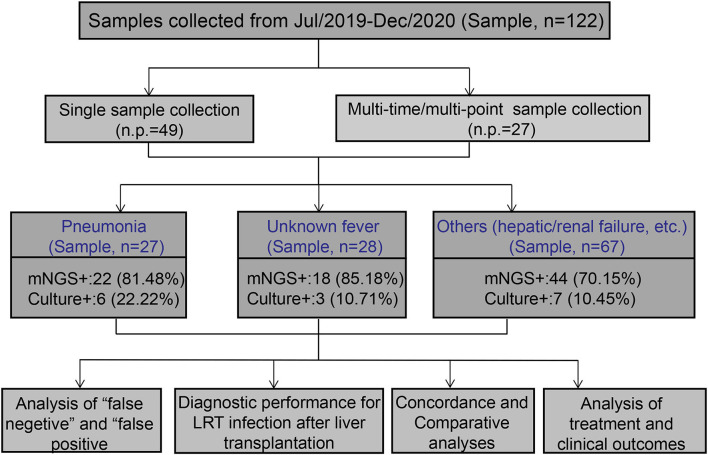
Flowchart of sample selection, classification, and comparison. A total of 122 samples were selected for further analysis. Samples were divided into pneumonia, unknown fever, and others groups based on the retrospective analysis of the corresponding patients. All samples were examined for the concordance analysis of metagenomic next-generation sequencing (mNGS) and culture technique. LRT, lower respiratory tract.

We retrospectively reviewed 122 clinical samples from patients with clinical suspicion of post-transplant infection at Shenzhen Third People's Hospital LT center between July 2019 and December 2020. In brief, these samples were obtained from 76 patients sampled at one/multiple sites at one/multiple time points (Figure 1). All patients had persistent fever or high fever, and all specimens were collected during fever. According to the possible reasons of fever and clinical symptoms and radiographic images, we divided the enrolled specimens into three groups. One was “pneumonia” group, that is, the clinical characteristics were consistent with the diagnostic indicators of pneumonia, such as cough, sputum, and pathological changes in CT slices with pneumonia. And one was suspected pneumonia group, which we also called “unknown fever” group. This group of patients has only fever, and no other symptoms of infection and no other complications are involved. Another was the “others” group, and this group of patients has obvious postoperative complications, such as liver failure, renal failure, and patients with severe rejection after liver transplantation. Considering that the cause of infection in patients with severe complications is usually organ failure caused by such complications, the main purpose of this study was to target the infection caused by non-complications after liver transplantation. So in this study, we did not subdivide the “others” group. All samples were subjected to routine clinical and microbiological assays and mNGS testing (BGI China), and pairwise comparisons were conducted.

### Sample Processing, DNA Extraction, Library Construction, and Sequencing

The samples included peripheral blood, alveolar lavage fluid (BALF), cerebrospinal fluid (CSF), ascites fluid, and sputum. About 3–4 mL of blood was taken from the patient and placed in an ethylenediaminetetraacetic acid tube for further treatment within 24 h. BALF was collected according to the standard procedure, that is, under aseptic conditions, a bronchoscope was inserted into the bronchus of the lesion site, and 100 ml sterile normal saline at 37°C was injected into the corresponding bronchus and sucked out under negative pressure (Fang et al., 2020). Specimens were stored in separate containers immediately after collection, except for sputum specimens, which were liquefied at room temperature with 0.1% DTT (dithiothreitol) for 30 min. What is noteworthy is that all the collected initial specimens were divided into two parts, one was used for subsequent mNGS sequencing, and the other was used for traditional culture. The standards and methods were implemented according to the routine microbial culture process, which was completed by the Clinical Laboratory of the Third People's Hospital of Shenzhen. Each of the samples, including the peripheral blood, BALF, CSF, ascites fluid, and sputum, was performed separately for laboratory culture.

For mNGS, 0.3 mL of the sample was separated into a new 1.5-mL microcentrifuge tube, and DNA was extracted using a TIANamp Micro DNA Kit (DP316, Tiangen Biotech) according to the manufacturer's instructions. Libraries were constructed using an end-repaired adapter, and polymerase chain reaction amplification of the extracted DNA was conducted. Quality control was carried out using a bioanalyzer (Agilent 2100, Agilent Technologies, Santa Clara, CA, USA) combined with PCR to measure the adapters before sequencing. Fifty bp single-end reads were then processed and DNA-sequenced using the BGISEQ-100 platform (BGI-Tianjin, Tianjin, China; Ming et al., 2016).

### Data Processing and Analysis

Sequencing data were generated by removing low-quality, and short (35 bp in length) reads, followed by computational subtraction of human host sequences, mapped to the human reference genome (hg38) using Burrows–Wheeler alignments. After removing low-complexity reads, data were classified by simultaneously comparing four microbial genome databases consisting of viruses, bacteria, fungi, and parasites. The taxonomic reference databases were downloaded from the National Center for Biotechnology Information (ftp://ftp.ncbi.nlm.nih.gov/genomes/). RefSeq contains 4,189 whole-genome sequences of viral taxa, 2,328 bacterial genomes or scaffolds, 199 fungi related to human infection, and 135 parasites associated with human diseases (Miao et al., 2018). The number of mapped reads, abundance, coverage rate, and species type at multiple points were used to identify positive infections.

### Criteria for a Positive Result

According to the clinical diagnostic standards of the clinical laboratory, pathogenic bacteria or fungi isolated and cultured in sterile body fluids (i.e., whole blood, CSF, ascites fluid) are usually regarded as the positive criterion, while the number of pathogenic bacteria or fungi in non-sterile body fluids (i.e., BALF, sputum) is often required to exceed 10^4^ cfu/mL to determine the positive. For mNGS, unique pathogenic reads were defined as reads whose alignment length was higher than 90%, identity with reference sequence higher than 95%, and the suboptimal to optimal alignment score ratio was lower than 0.8. The pathogen was further determined using the following criteria: (i) For absolutely sterile samples, such as blood, CSF, and ascites fluid, we set thresholds for viruses and fungi as > 20 reads or coverage > 1% or relative abundance > 15%, and thresholds for bacteria as coverage > 0.01%, unless there is a high abundance of reads in alveolar lavage fluid collected at the same point in time or blood cultures were positive; for non-strictly sterile samples, such as alveolar lavage fluid and sputum, the bacterial threshold was defined as coverage >0.2% or relative abundance >30% unless the culture results were positive; (ii) when mapping results showed different species of the same genus, the pathogen with the highest abundance was regarded as the positive species; (iii) if the microorganisms were only identified by mNGS, they were considered as new potential pathogens (Fang et al., 2020). Infectious pathogens were defined if any of the above conditions were met. One limitation of mNGS is the inability to establish whether the detected microorganisms were involved in infection, colonization, and contamination. Therefore, after obtaining the mNGS results, the clinical features were analyzed by two physicians, and a consensus was reached. In this study, the positive judgment was based on the results of mNGS and traditional microbial culture, as well as the clinical manifestations, that is, if any of the two methods were positive and consistent with clinical symptoms and laboratory findings, it was determined as a true infection.

### Statistical Analysis

Overall, the pathogen-positive rates among the groups and the species of pathogenic microorganisms among the groups were compared in detail, and the pathogen-positive rates for each type of samples were also statistically analyzed. In particular, pathogen detection rates in whole blood and BALF, as well as simple and mixed infections, were systematically analyzed. In addition, we also analyzed the sensitivity and specificity of mNGS and culture in the pneumonia group and the non-pneumonic fever group, that is, the unknown fever group. The paired McNemar chi-square test was used to compare the diagnostic efficiency of mNGS vs. conventional culture methods. Comparisons between groups were conducted by the chi-square test and Fisher's exact test (for discrete variables) where appropriate. A total of 2 × 2 contingency tables were derived to determine sensitivity, specificity, positive predictive value (PPV), and negative predictive value (NPV). The sensitivity and specificity were calculated based on the formula TP/(TP+FN) and TN/(TN+FP), respectively. PPV was expressed by the TP/(TP+FP) ratio, while NPV was calculated using TN/(TN+FN). Data analyses were performed using GraphPad Prism 9 software. P-values < 0.05 were statistically significant, and all tests were two-tailed.

## Results

### Patient Demographics and Clinical Features

In this study, a total of 122 samples from 76 patients treated with LT were analyzed (Figure 1). Sample acquisition consisted of different samples at multiple time points in 35.53% (27/76) of patients, while a single sample was collected for the remaining 49 patients. According to the clinical features, including the CT imaging results, we divided the samples into three groups, namely pneumonia (22.13%, *n* = 27), unknown fever (22.95%, *n* = 28), and others (i.e., hepatic failure, kidney failure, and postoperative examinations; 54.92%, *n* = 67) groups (Figure 1). The baseline characteristics of the patients, including gender, age, postoperative days, body temperature, C-reactive protein (CRP), procalcitonin (PCT), white blood cell (WBC), erythrocyte sedimentation rate (ESR), and the percentage of neutrophils, are listed in Supplementary Table 1.

### Comparison of the Diagnostic Performance of mNGS and Culture

**Figure 2:**
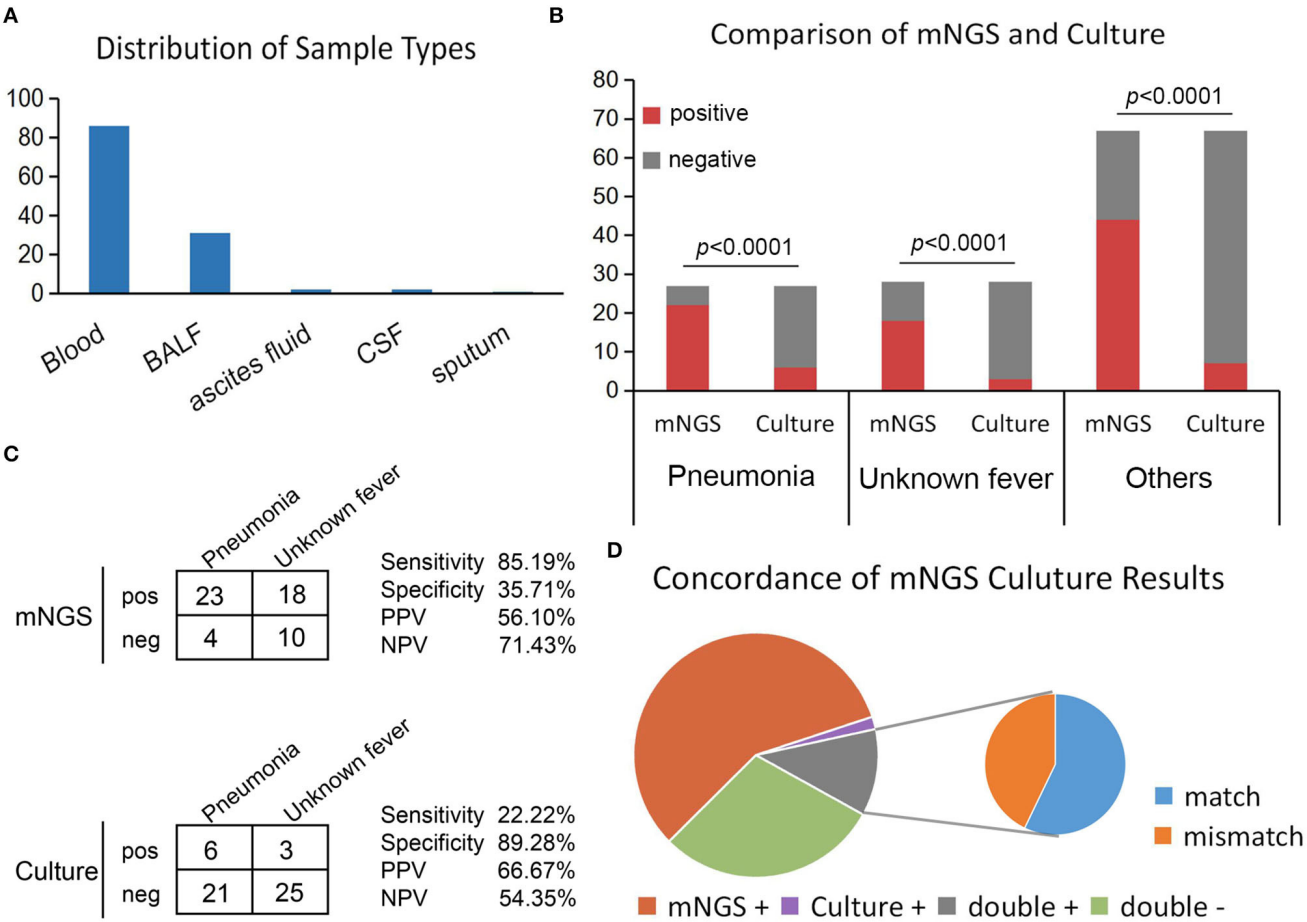
Positivity rate comparison and concordance analysis between mNGS and culture for infectious disease. **(A)** The number of samples involved in each sample type. **(B)** The number of positive samples (y-axis) for pairwise mNGS and culture testing is plotted against the pneumonia, unknown fever, and others groups (x-axis). **(C)** Contingency tables formatted in a 2×2 manner showing the respective diagnostic performance of mNGS and culture testing for differentiating pneumonia from unknown fever group. **(D)** Pie chart demonstrating the positivity distribution of mNGS and culture for all samples from three groups.

Overall, the sample types consisted of whole blood [*n* = 86 (70.49%)], followed by BALF [*n* = 31 (25.41%)], ascites fluid [*n* = 2 (1.64%)], cerebrospinal fluid [CSF, *n* = 2 (1.64%)], and sputum [*n* = 1 (0.81%)] (Figure 2A). Overall, the number of unique reads of the identified pathogens by mNGS ranged from 1 to 1,425,089. The coverage of identified pathogens ranged from 0.0004 to 100% with a depth value of 1–61.07, respectively. A comparison of the positivity rates of mNGS and culture tests (Figure 2B) showed that the mNGS positive detection rate was higher than culture in all groups (*p* < 0.0001). To compare the diagnostic efficiency in the pneumonia and unknown fever groups, 55 samples were included for further study. The negative and positive predictive values of diagnosing infectious disease by mNGS were 71.43 and 56.10%, with negative and positive likelihood ratios of 0.414 and 1.33, respectively. As expected, mNGS was associated with higher sensitivity (by ~63%; 85.19 vs. 22.22%; *p* < 0.0001), but lower specificity (35.71 vs. 89.28%; *p* < 0.0001) rates than pathogen culture (Figure 2C). Furthermore, in our results, mNGS and culture were both positive in 14 of 122 (11.48%) cases and were both negative in 36 of 122 (29.50%) cases. Positive findings were observed only in mNGS and culture tests in 57.38% (*n* = 70) and 1.64% (*n* = 2) of cases. For samples that yielded positive results for both approaches (*n* = 14), the results matched in eight cases (Figure 2D). The mNGS and culture results of the whole 122 samples in this study were shown in Supplementary Table 2.

### Infection Characteristics and Distribution of Pathogens After Liver Transplantation

**Figure 3:**
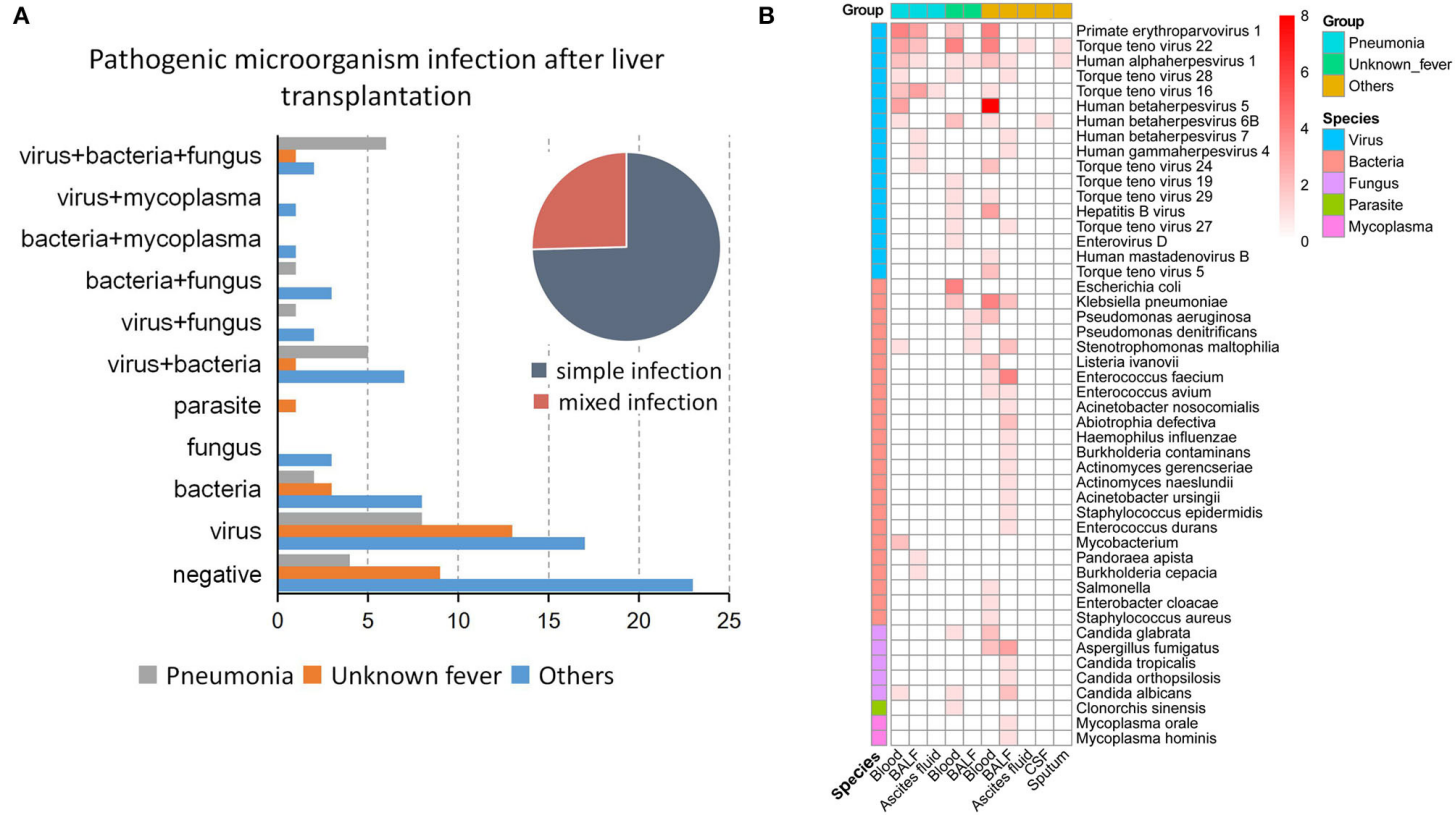
Types of infections and pathogen species after liver transplantation. **(A)** The type of infection in the three groups, including the proportion of simple and mixed infections. **(B)** The proportion of 48 pathogenic microorganisms in each sample type in the three groups.

In this study, we defined infection as microbial pathogens identified by either mNGS or culture methods and clinical symptoms as well as laboratory findings. Infectious complications after LT were divided into single and mixed infections, with the former accounting for about three-quarters of cases. For single infections, viruses [29.63% (8/27), 46.43% (13/28), and 29.82% (17/57) in the pneumonia, unknown fever, and others groups, respectively] represented the most common pathogens, followed by bacteria [7.40% (2/27), 10.71% (3/28), and 14.03 (8/57), respectively] (Figure 3A). In addition to single infections, our results showed that the LT patients were infected by various combinations of pathogens, such as virus, bacteria, and fungus co-infection, virus and bacteria co-infection, and bacteria and fungus co-infection (Figure 3A).

Among the 48 microbes identified, the Torque teno virus 22 (*n* = 15/122) was the most commonly detected pathogen, followed by Primate erythroparvovirus 1 (*n* = 13/122). The top four bacteria identified were *Klebsiella pneumonia* (*n* = 8), *Enterococcus faecium* (*n* = 5), *Stenotrophomonas maltophilia* (*n* = 4), and *Escherichia coli* (*n* = 4). Besides, Aspergillus fumigatus was the most commonly detected fungus in patients with postoperative liver transplant infection (Figure 3B). Notably, the pathogen species exhibited heterogeneous distributions in different groups and sample types. While only a single pathogen was identified in ascites fluid, CSF, and sputum, whole blood and BALF were associated with four to 24 pathogens (Figure 3B).

### Comparison of Pathogen Detection Rates in Whole Blood and BALF

**Figure 4:**
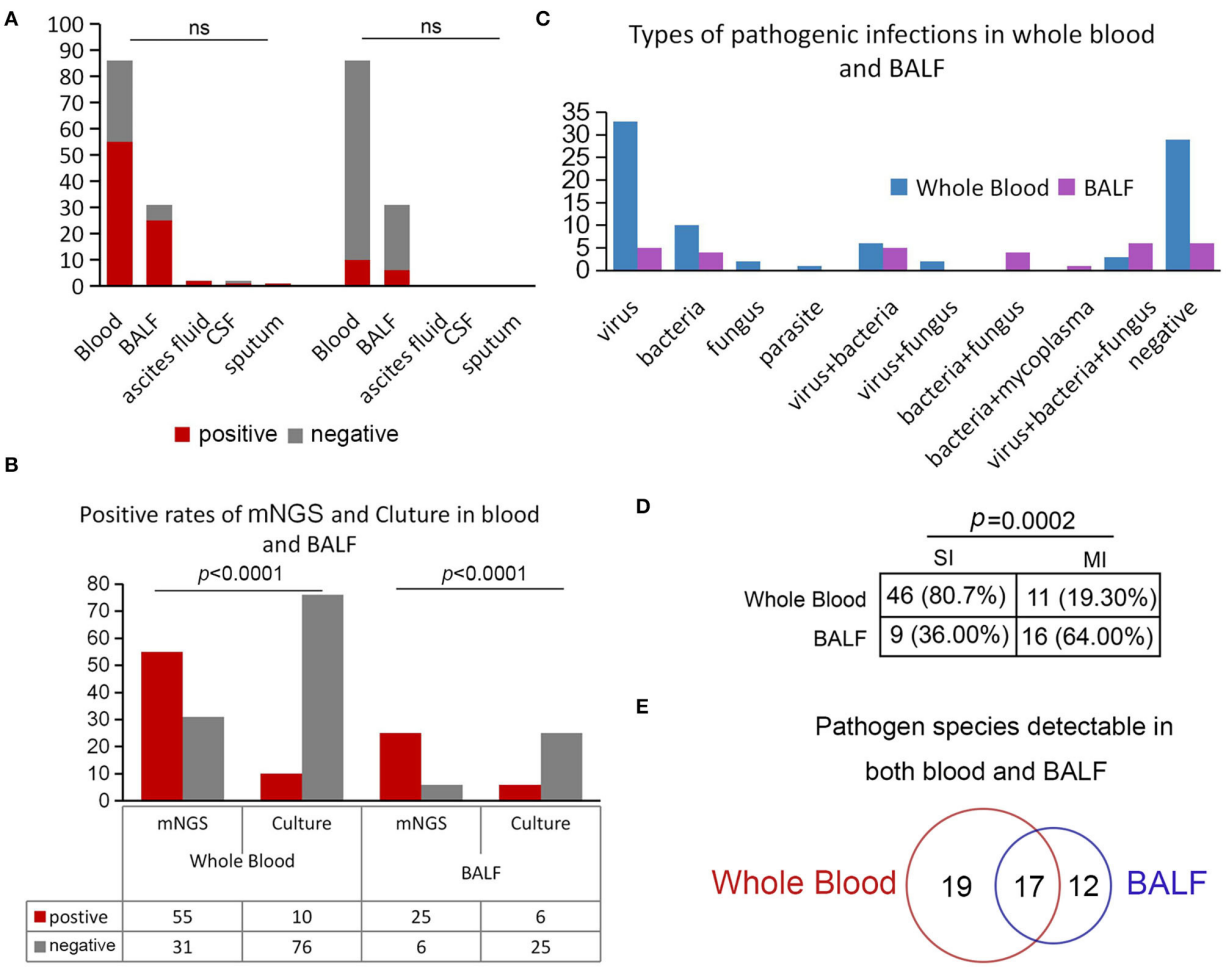
Positivity rate comparison and concordance analysis between whole blood and BALF. **(A)** Comparison of the positive rates of mNGS and culture in different sample types. **(B)** Positive rates of mNGS and culture in blood and BALF. **(C)** Positive rates of mNGS and culture in whole blood and BALF. **(D)** Comparison of simple and mixed infections in whole blood and BALF. **(E)** Pathogen species detected in both whole blood and BALF.

We also compared the positive rates between each sample type, and the results showed that the positive rates across different sample types of mNGS and culture had no difference (Figure 4A), whereas the positive detection rate by mNGS was significantly higher in whole blood and BALF samples compared to pathogen culture (*p* < 0.0001, Figure 4B). We further analyzed the types of pathogens in whole blood and BALF. The results showed that single viral infections were the most common type of infection in whole blood, with 38.37% (33/86) of samples exhibiting bloodstream infection, followed by bacteria (*n* = 10, 11.63%), fungi (*n* = 2, 2.33%), and parasites (*n* = 1, 1.16%; Figure 4C).

It is worth noting that mixed infections were more common in BALF, predominantly consisting of virus, fungus, and bacteria (*n* = 6, 19.35%), viral and bacterial co-infection (*n* = 5, 16.13%), or viral and fungal co-infection (*n* = 4, 12.90%) (Figure 4C). Among these positive samples, infections in BALF were complicated, with 64% (16/25) of samples with more than one type of pathogen in comparison with whole blood (*p* = 0.0002; Figure 4D). Notably, in our study, a total of 17 pathogens (Figure 4E), including eight kinds of viruses (i.e., Primate erythroparvovirus 1, Human alphaherpesvirus 1, Human betaherpesvirus 5, Human betaherpesvirus 6B, Torque teno virus 16, Torque teno virus 22, Torque teno virus 27, and Torque teno virus 29), five bacteria (i.e., *Klebsiella pneumoniae, Pseudomonas aeruginosa, Stenotrophomonas maltophilia, Enterococcus faecium*, and *Enterococcus avium*), and three kinds of fungi (i.e., *Aspergillus fumigatus, Candida orthopsilosis*, and *Candida albicans*), were detected both in BALF and whole blood, indicating that these pathogens could easily invade the blood and cause bloodstream infection after LT.

### Applications of Clinical mNGS Testing

Interestingly, the application of mNGS has assisted clinicians in diagnosing cases of unexplained fever after liver transplantation and optimizing anti-infection treatment strategies. Some cases are described below.

**Figure 5:**
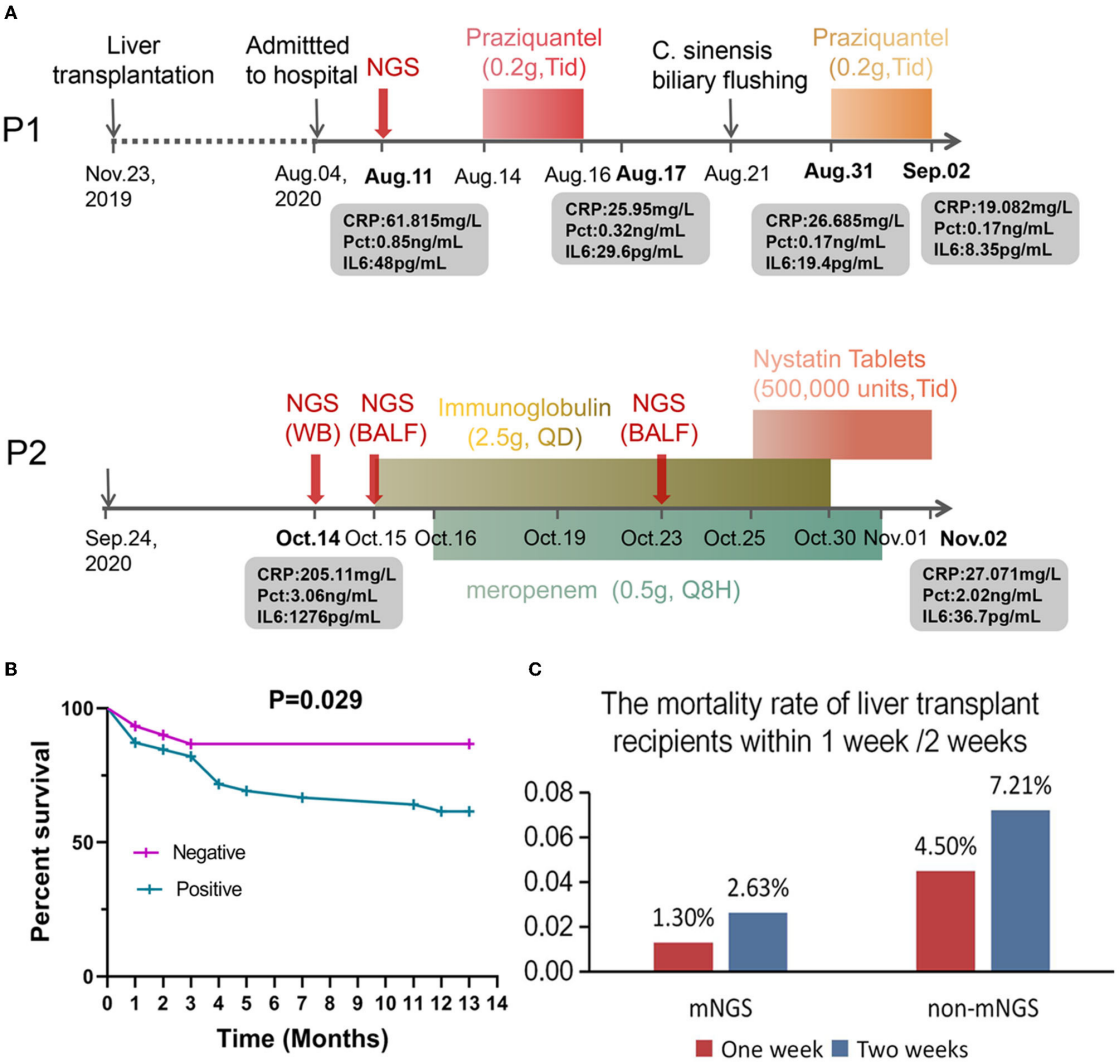
Applications of clinical mNGS testing and prognosis of patients after liver transplantation. **(A)** Application of mNGS to diagnose infection after liver transplantation and adjustment of drug according to mNGS results improved the patient's condition. **(B)** Analysis of mortality and survival in infected and uninfected patients. **(C)** The mortality rate of liver transplant recipients within 1 week /2 weeks.

Patient 1 is a 49-year-old male who underwent LT on November 23, 2019, and was admitted on August 4, 2020, complaining of recurrent hypothermia for 1 week (Figure 5A). The laboratory evaluation showed a high white blood cell count of 16.17 × 10^9^/L (normal, 3.5–9.5 × 10^9^/L), eosinophil percentage of 60.90% (normal, 0.4–8%), and low neutrophil percentage of 30.90% (normal, 40–75%). Despite several days of antibiotic treatment (Cefoperazone Sodium and Sulbactam Sodium, 1.5 g, Q8H), the laboratory examination on August 11 showed elevated C-reactive protein of 61.815 mg/L (CRP; normal, <10 mg/L), procalcitonin (Pct) of 0.85 ng/mL, and IL6 of 48 pg/mL (normal, 0–7 mg/mL). To determine the cause of the fever, mNGS was performed, and the next day's sequencing data showed 60 clonorchis sinensis sequences, while CDC did not report a positive trematode IGg until four days later. The patient was prescribed praziquantel (0.2 g, Tid) orally for 3 consecutive days from August 14 to 16 and from August 31 to September 2, respectively. On August 21, many Clonorchis Sinensis worms were flushed out of the biliary tract (Figure 5A). Subsequently, all blood indicators of the patient returned to normal, the patient's condition stabilized, and he was discharged 2 months later with a good prognosis.

Patient 2 is a 72-year-old male who underwent LT on September 24, 2020. Twenty days later, the patient exhibited fever, anorexia, and decreased blood pressure. The laboratory results upon admission showed high CRP (205.11 mg/L), PCT (3.06 ng/mL), and IL6 (1276 pg/mL) values, suggestive of an infection, but the BALF and whole blood culture were both negative. Subsequently, mNGS sequencing was performed on blood and BALF samples on October 14 and 15. The results showed that in addition to Klebsiella pneumoniae, the sequence number of human Primate erythroparvovirus 1 was also high (4,380 reads in blood and 633 reads in BALF). Immunoglobulin (2.5 g, QD) and meropenem (0.5 g, Q8H) were administered for infection control from October 15–October 30 and October 16–November 1, respectively. On October 23, mNGS was used to analyze the BALF sample of the patient. The sequencing results showed that the patient was infected with *Candida glabrata*, and Mycin tablets were prescribed for 7 days for antifungal treatment (Figure 5A). At the last follow-up, the patient was still alive.

We further analyzed the infection and survival of the patients within 1 week after liver transplantation (Table 1) and plotted the survival curve (Figure 5B). The 2019 “Expert Consensus on the Management of Metabolic Diseases in Chinese Liver Transplant Recipients” reported that the mortality rate of liver transplant recipients within 1 week was 2.2%, and the survival rate of patients in this study within 1 week after liver transplantation was 100%. In addition, among the 10 patients who were mNGS positive with or without positive cultures (Table 1), except for one patient who died 10 days after liver transplantation, the remaining patients survived for more than 3 months, suggesting that mNGS-assisted early diagnosis of infection after liver transplantation may effectively prolong the survival of this patient population. Notwithstanding that some infections were detected and treated in time due to mNGS, the overall fatality rate of the infected group was still ~3-fold higher than the non-infected group (13.33 vs. 38.46%, *p* = 0.029; Figure 5B), indicating that the prevention and diagnosis of infection after LT are equally important and require much emphasis. In order to compare the influence of mNGS on postoperative survival after liver transplantation, we retrospectively analyzed the mortality rates within 1 and 2 weeks of liver transplantation patients who did not receive mNGS-assisted diagnosis of infection during the same period (a total of 111 patients, and the details are shown in Supplementary Table 3) and compared them with the data of this study. The results showed that the 1-week and 2-week mortality rates were 1.30% (1/76) and 2.63% (2/76), respectively, in patients undergoing liver transplantation with mNGS and 4.5% (5/111) and 7.21%, respectively, in patients without mNGS (Figure 5C).

**Table 1:** Infection and survival data of patients 1 week after liver transplantation in this study.

**Patients**	**Gender**	**Age**	**Postoperative days**	**Infection**	**mNGS**	**Culture**	**Survival conditions**
1	Female	64	5	Yes	Human mastadenovirus B	-	Survived 187 days
2	Male	49	1	Yes	*Staphylococcus epidermidis, Enterococcus faecium, Candida albicans, Aspergillus fumigatus*	Aspergillus fumigatus	Survived
3	Male	47	7	No	-	-	Survived
4	Male	65	6	No	-	-	Survived
5	Female	36	3	Yes	Human betaherpesvirus 5, Torque teno virus 22, Primate erythroparvovirus 1	-	Survived
6	Male	47	2	No	-	-	Survived 14 days
7	Female	49	4	No	-	-	Survived
8	Male	55	3	No	-	-	Survived
9	Male	47	6	No	-	-	Survived 19 days
10	Male	51	4	No	-	-	Survived
11	Male	32	3	No	-	-	Survived
12	Male	70	3	No	-	-	Survived
13	Male	37	6	Yes	*Aspergillus fumigatus*	-	Survived
14	Male	37	2	No		-	Survived
15	Male	45	3	Yes	*Abiotrophia defectiva, Candida orthopsilosis*	-	Survived
16	Female	0+7	7	No	-	-	Survived
17	Male	68	4	Yes	*Klebsiella pneumoniae, Candida tropicalis*, Human gammaherpesvirus 4	*Candida tropicalis*	Survived 111 days
18	Male	34	3	Yes	virus+mycoplasma	-	Survived
19	Female	54	3	Yes	*Enterococcus faecium*	-	Survived
20	Male	52	4		-	-	Survived
21	Male	75	7	Yes	*Enterococcus avium*, Primate erythroparvovirus 1	Enterobacter cloacae	Survived
22	Male	42	5	No	-	-	Survived
23	Male	53	1	Yes	*Enterococcus faecium, Klebsiella pneumoniae, Aspergillus fumigatus*	*Klebsiella pneumoniae, Aspergillus fumigatus*	Survived 10 days
24	Male	53	3	No	-	-	Survived

*1. Negative results of mNGS or culture were marked as “-”*.

*2. A positive mNGS or culture that is consistent with clinical symptoms and laboratory results was defined as positive*.

## Discussion

In this study, we performed a hitherto undocumented comprehensive and systematic analysis of post-liver transplantation infectious complications diagnosed using mNGS and conducted pairwise comparisons with traditional culture. The results showed that postoperative infection was a common complication after LT, and more than 70% of the samples in this study showed single or mixed infection, consistent with previous reports that infection occurs in up to 80% of the orthotopic LT patients (Hoek et al., 2012).

Moreover, the mNGS positive detection rate was higher than pathogen culture in all groups (*p* < 0.0001), indicating that the rapid mNGS testing improved clinical decision-making and guided treatment selection. However, a comparison between pneumonia and unknown fever groups found that mNGS had a higher sensitivity (85.19 vs. 22.22%) but lower specificity (35.71 vs. 89.28%). In addition, for specimens that yielded positive results with both methods, although most mNGS results corresponded to the pathogen type identified by culture, there were some inconsistencies, suggesting that the final diagnosis of infection after LT requires the combination of both methods.

Growing evidence suggests that infectious diseases after solid organ transplantation (SOT) are a significant cause of morbidity and reduced allograft and patient survival (Hoek et al., 2012; Martin-Gandul et al., 2015). Owing to the complexity of LT and the high susceptibility of these patients, the risk factors associated with post-LT pneumonia and other infections are numerous (Angarita et al., 2017). Interestingly, it has been reported that within 1 month after LT, Clostridium difficile colitis, herpes, and Candida infections are dominant, while opportunistic infections, such as CMV (cytomegalovirus, also known as Human betaherpesvirus 5) and EBV (Epstein–Barr virus, also known as Human herpesvirus 4), are predominantly observed at 1–6 months after LT. Beyond 6 months, most infections are community-acquired and Hepatitis C virus recurrence (Hoek et al., 2012). However, to the best of our knowledge, mNGS is rarely used during clinical practice for the etiological diagnosis and rational use of antibiotics in cases of post-transplant infection. In recent years, mNGS has been used to diagnose early human parvovirus (HPV) B19 infection after liver transplantation, suggesting that mNGS is an effective method for early disease screening (Chen et al., 2019). Our study suggested that, in addition to CMV, the Torque Teno virus (TTV) is a common infectious agent in patients after LT. Interestingly, Torque teno virus (TTV) was the first human Anelloviridae detected in HIV/HBV patients, chronic hepatitis B patients, and healthy individuals (Najafimemar et al., 2017; Faab et al., 2020). At present, the underlying pathogenesis remains poorly understood, nor is the relationship between infection and patient outcome after LT clear. Interestingly, whole blood may be an appropriate sample type for detecting viral infection. It is widely acknowledged that some viral infections can easily enter the blood and cause viremia. Nonetheless, it should be borne in mind that compared with pathogen culture (Rhodes et al., 2010), the survival time of pathogenic DNA in plasma is longer (Gosiewski et al., 2017), which is conducive to its clinical application for diagnosing complex infectious diseases. The turnaround time in our cohort was ~24 h for DNA-Seq and could be reduced to <20 h soon due to the establishment of a sequencing platform at our hospital. In contrast, the average feedback time of pathogen culture is ≥3 days for bacteria and 7 days for fungi (Miao et al., 2018). In addition, there is a paucity of laboratory methods to detect parasites and viruses since all species cannot be covered. Intriguingly, many cases of mNGS-assisted pathogen detection have been reported in other types of infection, such as sepsis, bacterial meningitis, and immunocompromised people (Parize et al., 2017; Blauwkamp et al., 2019; Wilson et al., 2019). This study provided compelling evidence of the effective use of mNGS clinically, with several cases of infection with negative cultures that were confirmed by mNGS or with earlier results for mNGS than traditional culture in some cases. Importantly, we successfully identified a case of Clonorchis Sinensis infection using mNGS and treated the patient with appropriate anti-parasitic therapy. To our knowledge, this is the first study to analyze pneumonia and other causes of infection using mNGS in post-LT patients.

Admittedly, there were several limitations in our research. Although our primary task was to diagnose pneumonia after LT, and a significant proportion of the samples analyzed were whole blood samples, it was difficult to establish whether the primary source of bloodstream infection was pulmonary inflammation without simultaneous BALF collection. Furthermore, our non-targeted mNGS approach was not comprehensive enough. For example, DNA and RNA sequencing were not conducted simultaneously, which could have led to a lack of valuable complementary information such as RNA virus and microbial transcriptome changes. Besides, due to the lack of negative controls in healthy people in this study, we could only judge which samples were positive according to the pathogen abundance, although colonization and contamination could not be completely ruled out. Indeed, the interpretation of mNGS results may be challenging when sequence reads of commensal organisms are aligned with those of multiple potential pathogens (Haslam, 2021). In addition, the existing results of this study could not fully explain the mismatching of some pathogen species identified by mNGS and traditional culture. Generally speaking, microbial culture is universally recognized as the gold standard. It remains unclear whether some pathogens could not be identified due to the insufficient depth of mNGS sequencing. Finally, although the mortality rates of patients in the non-mNGS group were 4.5 and 7.21% within 1 and 2 weeks after surgery, respectively, while in the mNGS group were 1.3 and 4.5%, respectively, due to the limited number of samples in this study and many causes of death within 2 weeks after liver transplantation, such as arterial blockage, therefore, it is not clear whether the mortality is directly related to the use of mNGS. However, high-throughput sequencing, including mNGS, has huge prospects for application in clinical diagnosis, especially for complex infections after LT. Further studies are warranted.

In conclusion, our study explored the application of mNGS in pneumonia and other infections in patients after LT for the first time and conducted a systematic cross-sectional study on infections after LT. The results suggested that the Torque teno virus 22, Klebsiella pneumoniae, and the Aspergillus fumigatus are the most common pathogens of viruses, bacteria, and fungi after LT, suggesting that these pathogens may be associated with postoperative pathogen infection and patient prognosis. The mNGS technology brings many advantages in detecting opportunistic pathogens and mixed infections in this patient population. Besides, our results suggest that using mNGS to diagnose infection after early liver transplantation may effectively prolong patient survival. Importantly, we provide compelling evidence that mNGS technology enables timely detection and treatment of infections since mortality of the infected group after LT was significantly higher than the non-infected group, suggesting that prevention and diagnosis of infection after LT are equally important. Further studies are warranted to explore how to harness the advantages of high-throughput technology to clarify the dynamic evolution of infection and molecular characteristics after transplantation, identify risk biomarkers for post-transplantation infection, and predict critical illness.

## Data Availability Statement

The original contributions presented in the study are included in the article/Supplementary Material, further inquiries can be directed to the corresponding author.

## Ethics Statement

The studies involving human participants were reviewed and approved by Shenzhen Third People's Hospital, Second Affiliated Hospital, Southern University of Science and Technology (Approved Number: 2022-037-02). Written informed consent to participate in this study was provided by the participants' legal guardian/next of kin.

## Author Contributions

JY, DZ, and LG conceptualized and designed this study. DZ and LG performed data analysis and drafted the paper. DL, YG, and XY collected clinical data. HH and JY revised the manuscript. All authors contributed to the article and approved the submitted version.

## Funding

This study was supported in part by Shenzhen Fund for Guangdong Provincial High-Level Clinical Key Specialties (No. SZGSP011), the 14th Five-Year Plan National Key R&D Project: Research on the mechanism of critical illness caused by cytokine storm (No. 2021YFC2301803), Shenzhen Key Medical Discipline Construction Fund (No. SZXK079), Science and Technology Program of Shenzhen, China (No. JCYJ20170307095236069), and the clinical research project of Shenzhen Third People's Hospital (No. G2021008 and G2022008).

## Conflict of Interest

The authors declare that the research was conducted in the absence of any commercial or financial relationships that could be construed as a potential conflict of interest.

## Publisher's Note

All claims expressed in this article are solely those of the authors and do not necessarily represent those of their affiliated organizations, or those of the publisher, the editors and the reviewers. Any product that may be evaluated in this article, or claim that may be made by its manufacturer, is not guaranteed or endorsed by the publisher.

## Supplementary Material

Supplementary Table 1 Baseline characteristics of patients in this study.

Supplementary Table 2 Metagenomic next-generation sequencing and culture results of the whole 122 samples in this study.

Supplementary Table 3 Survival of patients who did not receive mNGS.
