# Supplementary material for: Diagnostic Value and Clinical Application of mNGS for Post-Liver Transplantation Infection: A Cross-Sectional Study With Case Reports
Source: Front Microbiol. 2022 Jul 1;13:919363. doi: 10.3389/fmicb.2022.919363 (PMC9283086; doi:10.3389/fmicb.2022.919363)
Supplement: Supplementary Table 1 — Baseline characteristics of patients in this study. [file Table_1.DOC]

| Sample | Gender | Age | Postoperative days | body temperature  （℃） | CRP  （mg/L） | PCT  （ng/mL） | WBC （10^9/L） | ESR （mm/h） |
| --- | --- | --- | --- | --- | --- | --- | --- | --- |
| 1 | Male | 39 | 341 | 38.8 | 197.67 | 0.082 | 5.53 | 115 |
| 2 | Male | 39 | 353 | 38.5 | 66.611 | 3.65 | 2.73 | 130 |
| 3 | Male | 46 | 278 | 39.5 | 45.828 | 33.5 | 20.93 | 84 |
| 4 | Male | 62 | 27 | 39.7 | 49.65 | 1.85 | 2.65 | 12 |
| 5 | Male | 51 | 31 | 36.7 | 193.4 | 0.2017 | 19.93 |  |
| 6 | Male | 49 | 65 | 38.9 | 17.62 | 0.093 | 2.56 | 48 |
| 7 | Male | 50 | 524 | 37.1 | 11.578 | 0.04 | 7.97 | 44 |
| 8 | Famale | 64 | 5 | 38.8 | 80.03 | 1.73 | 2.79 |  |
| 9 | Male | 49 | 1 | 38.5 | 20.23 | 3.02 | 11.96 | 15 |
| 10 | Famale | 43 | 59 | 38.3 | 38.601 | 0.74 | 10.97 | 19 |
| 11 | Male | 47 | 7 | 38 | 49.8 | 1.64 | 3.27 | 8 |
| 12 | Famale | 67 | 69 | 38.8 | 122.9 | 0.32 | 4.75 |  |
| 13 | Famale | 67 | 75 | 38.3 | 184.98 | 0.787 | 10.31 |  |
| 14 | Famale | 7 | 14 | 38.6 | 33.583 | 0.29 | 11.14 |  |
| 15 | Famale | 8 | 83 |  | 34.666 | 0.19 | 2.23 | 10 |
| 16 | Male | 65 | 6 | 38.8 | 36.79 | 3.32 | 2.78 | 7 |
| 17 | Famale | 36 | 3 | 38 | 42.38 | 5.01 | 8.08 | 45 |
| 18 | Male | 54 | 8 | 38.3 | 66.52 | 1.25 | 7.27 |  |
| 19 | Famale | 69 | 9 | 38 | 65.525 | 1.08 | 5.5 | 40 |
| 20 | Famale | 69 | 27 | 39 | 207.6 | 1.243 | 5.64 |  |
| 21 | Famale | 69 | 32 | 38.5 | 58.271 | 0.9 | 4.38 |  |
| 22 | Famale | 69 | 35 | 36.9 | 43.071 | 0.25 | 3.37 |  |
| 23 | Male | 72 | 20 | 37.4 | 205.11 | 3.06 | 11.24 | 26 |
| 24 | Male | 72 | 21 | 36.8 | 247.08 | 4.39 | 9.97 |  |
| 25 | Male | 72 | 29 | 37.1 | 69.862 | 3.87 | 12.84 |  |
| 26 | Male | 49 | 24 | 39.4 | 24.766 | 1.06 | 4.35 | 13 |
| 27 | Male | 49 | 73 | 38.5 | 54.5 | 0.4045 | 2.18 |  |
| 28 | Male | 55 | 9 | 36.9 | 48.521 | 3.71 | 7.69 |  |
| 29 | Male | 47 | 2 | 36.5 | 1.86 | 0.153 | 6.61 |  |
| 30 | Male | 51 | 12 | 39.3 | 99.91 | 0.62 | 6.6 |  |
| 31 | Famale | 41 | 23 | 39.2 | 92.4 | ＞100 | 1.16 |  |
| 32 | Famale | 49 | 4 | 36.7 | 7.122 | 3.45 | 5.1 |  |
| 33 | Male | 41 | 104 | 37 | 26.1 | 0.26 | 3.61 |  |
| 34 | Male | 56 | 25 | 38.5 | 37.78 | 0.681 | 13.64 |  |
| 35 | Male | 57 | 36 | 36.9 | 55.16 | 0.521 | 5.21 | 108 |
| 36 | Famale | 66 | 49 | 38 | 34.271 | 0.27 | 5.94 |  |
| 37 | Male | 55 | 3 | 39.8 | 38.739 | 0.31 | 8.08 |  |
| 38 | Male | 55 | 34 | 36.8 | 46.262 | 0.07 | 2.8 |  |
| 39 | Male | 51 | 10 | 37.4 | 8.572 | 0.25 | 4.51 |  |
| 40 | Male | 51 | 31 | 38.8 | 132.7 | 0.1 | 2.6 | 54 |
| 41 | Male | 47 | 6 | 38 | 53.154 | 0.79 | 4.3 |  |
| 42 | Male | 51 | 4 | 37.4 | 12.37 | 0.663 | 10.51 |  |
| 43 | Male | 51 | 8 | 39.7 | 19.65 | 0.174 | 11.23 |  |
| 44 | Male | 1 | 22 | 37.5 | 7.116 | 0.38 | 4.99 |  |
| 45 | Male | 1 | 51 | 39.8 | 54 | 1.23 | 16.94 |  |
| 46 | Male | 32 | 3 | 39.2 | 9.187 | 1.16 | 10.21 |  |
| 47 | Male | 62 | 88 | 37.2 | 119.93 | 0.985 | 4.41 |  |
| 48 | Male | 46 | 252 | 38.3 | 32.034 | 0.1 | 7.49 | 97 |
| 49 | Male | 29 | 170 | 38.7 | 54.73 | 0.241 | 3.13 | 37 |
| 50 | Famale | 1 | 10 | 39.3 | 63.746 | 2.19 | 16.35 |  |
| 51 | Male | 49 | 54 | 37 | 79.9 | 0.63 | 14.39 |  |
| 52 | Male | 49 | 56 | 37.8 | 21.703 | 0.1 | 5.93 |  |
| 53 | Male | 53 | 19 | 42 | 81.804 | 20.7 | 7.54 |  |
| 54 | Male | 70 | 3 | 39.4 | 33.4 |  | 5.53 |  |
| 55 | Male | 37 | 6 | 38.4 | 11.187 | 3.19 | 14.02 |  |
| 56 | Famale | 44 | 17 | 37.8 | 26.56 | 4.14 | 11.52 |  |
| 57 | Famale | 44 | 23 | 39.1 | 38.58 | 3.76 | 7.23 |  |
| 58 | Famale | 44 | 116 | 38.4 | 30.929 | 0.55 | 3.04 |  |
| 59 | Famale | 45 | 238 | 37.5 | 141.13 | 47.4 | 38.31 |  |
| 60 | Male | 37 | 2 | 38.1 | 12.594 | 14.9 | 7.78 |  |
| 61 | Male | 37 | 8 | 36.9 | 0.976 | 0.38 | 4.53 |  |
| 62 | Male | 45 | 3 | 37.3 | 52.758 | 0.8 | 7.52 |  |
| 63 | Male | 46 | 40 | 39.5 | 91.249 | 0.18 | 1.11 |  |
| 64 | Famale | 58 | 13 | 38.4 | 7.01 | 0.475 | 18.6 | 18 |
| 65 | Male | 55 | 8 | 38.6 | 50.505 | 1.33 | 10.47 | 7 |
| 66 | Male | 55 | 80 | 38.1 | 70.894 | 1.08 | 3.87 |  |
| 67 | Male | 55 | 81 | 37.1 | 91.306 | 0.49 | 4.75 |  |
| 68 | Famale | 0+7 | 7 | 38.5 | 89.802 | 0.98 | 22.23 |  |
| 69 | Male | 68 | 4 | 36.8 | 18.069 | 4.26 | 4.34 | 5 |
| 70 | Male | 68 | 8 | 36.9 | 30.86 | 1.89 | 5.48 |  |
| 71 | Male | 68 | 33 | 36.8 | 166.31 | 20.7 | 4.74 |  |
| 72 | Male | 68 | 35 | 40.1 | 124.2 | ＞100 | 25.77 |  |
| 73 | Male | 48 | 43 | 37.2 | 161.88 | 1.13 | 7.12 | 77 |
| 74 | Male | 48 | 44 | 37 | 85.135 | 0.71 | 7.26 | 95 |
| 75 | Male | 34 | 3 | 37 | 12.22 | 3.28 | 8.55 |  |
| 76 | Famale | 54 | 3 | 37.7 | 41.04 | 2.01 | 4.87 |  |
| 77 | Male | 52 | 4 | 36.5 | 34.412 | 4.42 | 6.15 |  |
| 78 | Male | 52 | 13 | 37.9 | 100.52 | 2.42 | 4.69 | 24 |
| 79 | Male | 47 | 116 | 36.7 | 59.56 | 0.304 | 15.89 |  |
| 80 | Male | 47 | 118 | 37.2 | 70.34 | 0.209 | 5.16 |  |
| 81 | Male | 47 | 126 | 37 | 226.85 | 1 | 3.84 |  |
| 82 | Male | 57 | 11 | 37.5 | 30.61 | 2 | 15.99 |  |
| 83 | Famale | 41 | 10 | 38.5 | 33.51 | 0.08 | 7.04 | 19 |
| 84 | Famale | 41 | 21 | 38.2 | 44.429 | 0.04 | 12.49 |  |
| 85 | Famale | 41 | 23 | 37.4 | 37.554 | 0.03 | 6.13 | 55 |
| 86 | Male | 63 | 8 | 38.4 | 79.722 | 0.73 | 5.37 | 85 |
| 87 | Male | 63 | 11 | 38 | 40.35 | 0.64 | 3.07 |  |
| 88 | Male | 64 | 151 | 36.8 | 67.2 | 0.72 | 2.41 |  |
| 89 | Male | 68 | 126 | 39 | 162.77 | 2.07 | 6.07 | 95 |
| 90 | Male | 63 | 8 | 38.3 | 24.558 | 0.63 | 5.73 | 5 |
| 91 | Male | 75 | 7 | 38.6 | 70.542 | 0.97 | 5.72 |  |
| 92 | Male | 64 | 8 | 37 | 15.911 | 0.93 | 5.35 | 2 |
| 93 | Male | 42 | 5 | 39.2 | 39.594 | 1.02 | 2.77 |  |
| 94 | Male | 61 | 42 | 37.3 | 1.581 | 0.17 | 16.02 |  |
| 95 | Famale | 63 | 8 | 39.2 | 44.72 | 0.431 | 6.96 | 30 |
| 96 | Male | 49 | 262 | 37.5 | 61.815 | 0.85 | 39.47 |  |
| 97 | Male | 47 | 21 | 37.8 | 69.69 | 0.309 | 4.28 | 98 |
| 98 | Famale | 53 | 97 | 39.3 | 131.31 | 0.65 | 7.56 |  |
| 99 | Male | 54 | 10 | 39 | 118.25 | 0.37 | 5 |  |
| 100 | Male | 53 | 1 | 39 | 95.5 | ＞100 | 15.44 |  |
| 101 | Male | 53 | 3 | 36.5 | 135.14 | 32.24 | 8.04 |  |
| 102 | Male | 53 | 3 | 38.9 | 10.859 | 1.43 | 3.02 | 8 |
| 103 | Male | 53 | 80 | 38.5 | 213.23 | 2.4 | 10.16 | 111 |
| 104 | Male | 53 | 86 | 38 | 121.62 | 0.28 | 4.98 |  |
