# Supplementary material for: Diagnostic Value and Clinical Application of mNGS for Post-Liver Transplantation Infection: A Cross-Sectional Study With Case Reports
Source: Front Microbiol. 2022 Jul 1;13:919363. doi: 10.3389/fmicb.2022.919363 (PMC9283086; doi:10.3389/fmicb.2022.919363)
Supplement: Supplementary Table 3 — Survival of patients who did not receive mNGS. [file Table_3.DOCX]

| Patients | Gender | Age | Date of liver transplantation | Survival conditions | Date of death after liver transplantation |
| --- | --- | --- | --- | --- | --- |
| P1 | Male | 76 | 2019/7/8 | Died in 2020/4/20 | 287 |
| P2 | Male | 59 | 2019/7/31 | Died in 2022/1/14 | 898 |
| P3 | Famale | 46 | 2019/8/2 |  |  |
| P4 | Male | 36 | 2019/8/4 |  |  |
| P5 | Male | 42 | 2019/8/6 |  |  |
| P6 | Male | 57 | 2019/8/8 | Died in 2019/8/9 | 1 |
| P7 | Male | 52 | 2019/8/8 |  |  |
| P8 | Male | 42 | 2019/8/9 |  |  |
| P9 | Male | 69 | 2019/8/11 |  |  |
| P10 | Male | 57 | 2019/8/11 |  |  |
| P11 | Male | 38 | 2019/8/13 |  |  |
| P12 | Male | 37 | 2018/8/18 |  |  |
| P13 | Male | 56 | 2019/8/19 |  |  |
| P14 | Famale | 50 | 2019/8/24 | Died in 2019/9/24 | 31 |
| P15 | Male | 45 | 2019/8/24 |  |  |
| P16 | Male | 30 | 2019/8/29 |  |  |
| P17 | Male | 54 | 2019/9/11 |  |  |
| P18 | Male | 17 | 2019/9/19 |  |  |
| P19 | Male | 47 | 2019/9/20 |  |  |
| P20 | Male | 48 | 2019/9/20 |  |  |
| P21 | Male | 47 | 2019/9/21 |  |  |
| P22 | Male | 48 | 2019/9/25 |  |  |
| P23 | Male | 47 | 2019/9/25 | Died in 2019/9/27 | 2 |
| P24 | Famale | 67 | 2019/9/28 |  |  |
| P25 | Male | 33 | 2019/10/1 |  |  |
| P26 | Male | 47 | 2019/10/9 |  |  |
| P27 | Male | 46 | 2019/10/12 |  |  |
| P28 | Famale | 52 | 2019/10/13 |  |  |
| P29 | Male | 69 | 2019/10/14 |  |  |
| P30 | Male | 33 | 2019/10/27 |  |  |
| P31 | Male | 54 | 2019/11/7 |  |  |
| P32 | Male | 37 | 2019/11/9 |  |  |
| P33 | Famale | 60 | 2019/11/14 |  |  |
| P34 | Famale | 62 | 2019/11/17 | Died in 2020/6/16 | 212 |
| P35 | Male | 46 | 2019/11/19 |  |  |
| P36 | Male | 36 | 2019/11/20 |  |  |
| P37 | Male | 31 | 2019/11/27 |  |  |
| P38 | Male | 31 | 2019/12/13 |  |  |
| P39 | Male | 31 | 2019/12/27 |  |  |
| P40 | Male | 60 | 2019/12/28 |  |  |
| P41 | Male | 38 | 2020/1/2 |  |  |
| P42 | Male | 38 | 2020/1/4 | Died in 2020/1/13 | 9 |
| P43 | Male | 52 | 2020/1/8 | Died in 2020/2/2 | 25 |
| P44 | Male | 40 | 2020/1/19 | Died in 2020/1/21 | 2 |
| P45 | Male | 48 | 2020/1/20 | Died in 2020/1/31 | 11 |
| P46 | Male | 34 | 2020/1/23 |  |  |
| P47 | Male | 52 | 2020/1/23 |  |  |
| P48 | Male | 46 | 2020/1/24 |  |  |
| P49 | Male | 43 | 2020/2/2 |  |  |
| P50 | Male | 32 | 2020/2/3 |  |  |
| P51 | Male | 40 | 2020/2/15 |  |  |
| P52 | Male | 46 | 2020/2/24 | Died in 2020/11/27 | 277 |
| P53 | Male | 50 | 2020/2/29 | Died in 2020/3/6 | 6 |
| P54 | Male | 57 | 2020/3/2 | Died in 2020/3/3 | 1 |
| P55 | Male | 30 | 2020/3/8 |  |  |
| P56 | Male | 51 | 2020/3/11 |  |  |
| P57 | Famale | 63 | 2020/4/7 |  |  |
| P58 | Male | 50 | 2020/4/9 | Died in 2020/10/22 | 196 |
| P59 | Famale | 29 | 2020/4/21 |  |  |
| P60 | Male | 31 | 2020/5/13 |  |  |
| P61 | Male | 70 | 2020/5/13 | Died in 2020/6/10 | 28 |
| P62 | Male | 27 | 2020/5/17 |  |  |
| P63 | Male | 47 | 2020/5/20 |  |  |
| P64 | Male | 46 | 2020/5/21 | Died in 2020/6/2 | 12 |
| P65 | Male | 48 | 2020/5/22 |  |  |
| P66 | Male | 51 | 2020/5/29 |  |  |
| P67 | Male | 62 | 2020/6/11 | Died in 2020/7/11 | 30 |
| P68 | Male | 51 | 2020/6/16 | Died in 2020/7/16 | 30 |
| P69 | Male | 50 | 2020/6/24 |  |  |
| P70 | Male | 48 | 2020/6/28 |  |  |
| P71 | Male | 30 | 2020/7/1 |  |  |
| P72 | Male | 63 | 2020/7/3 |  |  |
| P73 | Male | 13 | 2020/7/4 | Died in 2022/1/1 | 546 |
| P74 | Famale | 69 | 2020/7/8 |  |  |
| P75 | Male | 59 | 2020/7/10 |  |  |
| P76 | Male | 48 | 2020/8/6 |  |  |
| P77 | Male | 48 | 2020/8/8 |  |  |
| P78 | Male | 59 | 2020/8/13 | Died in 2021/06 |  |
| P79 | Male | 38 | 2020/8/18 |  |  |
| P80 | Male | 1 | 2020/8/18 |  |  |
| P81 | Male | 51 | 2020/8/19 | Died in 2020/9/5 | 17 |
| P82 | Male | 30 | 2020/8/20 |  |  |
| P83 | Famale | 55 | 2020/8/23 |  |  |
| P84 | Male | 38 | 2020/8/25 | Died in 2020/12/24 | 121 |
| P85 | Male | 43 | 2020/8/26 |  |  |
| P86 | Male | 45 | 2020/9/4 |  |  |
| P87 | Male | 56 | 2020/9/5 |  |  |
| P88 | Famale | 66 | 2020/9/16 |  |  |
| P89 | Male | 28 | 2020/9/20 |  |  |
| P90 | Male | 61 | 2020/9/29 | Died in 2020/12/3 | 65 |
| P91 | Famale | 36 | 2020/9/30 |  |  |
| P92 | Male | 45 | 2020/10/4 | Died in 2020/10/22 | 18 |
| P93 | Famale | 31 | 2020/10/5 |  |  |
| P94 | Male | 47 | 2020/10/6 |  |  |
| P95 | Male | 59 | 2020/10/9 |  |  |
| P96 | Male | 31 | 2020/10/23 | Died in 2021/6/25 | 245 |
| P97 | Male | 59 | 2020/10/28 |  |  |
| P98 | Male | 43 | 2020/11/1 |  |  |
| P99 | Male | 50 | 2020/11/10 | Died in 2020/12/5 | 25 |
| P100 | Male | 37 | 2020/11/14 |  |  |
| P101 | Male | 61 | 2020/11/15 |  |  |
| P102 | Famale | 68 | 2020/11/19 | Died in 2021/5/10 | 172 |
| P103 | Male | 45 | 2020/11/30 |  |  |
| P104 | Male | 46 | 2020/12/12 |  |  |
| P105 | Male | 37 | 2020/12/13 | Died in 2021/1/7 | 25 |
| P106 | Male | 45 | 2020/12/13 |  |  |
| P107 | Male | 65 | 2020/12/14 |  |  |
| P108 | Male | 56 | 2020/12/15 |  |  |
| P109 | Male | 34 | 2020/12/19 | Died in 2021/5/25 | 157 |
| P110 | Male | 51 | 2020/12/23 |  |  |
| P111 | Male | 39 | 2020/12/24 | Died in 2021/1/12 | 19 |
